# An original *SERPINA3 *gene cluster: Elucidation of genomic organization and gene expression in the *Bos taurus *21q24 region

**DOI:** 10.1186/1471-2164-9-151

**Published:** 2008-04-02

**Authors:** Patrick Pelissier, Didier Delourme, Agnes Germot, Xavier Blanchet, Samira Becila, Abderrahman Maftah, Hubert Leveziel, Ahmed Ouali, Laure Bremaud

**Affiliations:** 1 INRA, UMR 1061 Unité de Génétique Moléculaire Animale, Université de Limoges, IFR 145, Faculté des Sciences et Techniques, 87060 Limoges, France; 2 QuaPA, BPM, INRA de Clermont Ferrand-Theix, 63122 Saint Genes Champanelle, France

## Abstract

**Background:**

The superfamily of ***ser***ine ***p***roteinase ***in***hibitors (serpins) is involved in numerous fundamental biological processes as inflammation, blood coagulation and apoptosis. Our interest is focused on the SERPINA3 sub-family. The major human plasma protease inhibitor, α1-antichymotrypsin, encoded by the *SERPINA3 *gene, is homologous to genes organized in clusters in several mammalian species. However, although there is a similar genic organization with a high degree of sequence conservation, the reactive-centre-loop domains, which are responsible for the protease specificity, show significant divergences.

**Results:**

We provide additional information by analyzing the situation of *SERPINA3 *in the bovine genome. A cluster of eight genes and one pseudogene sharing a high degree of identity and the same structural organization was characterized. Bovine *SERPINA3 *genes were localized by radiation hybrid mapping on 21q24 and only spanned over 235 Kilobases. For all these genes, we propose a new nomenclature from *SERPINA3-1 *to *SERPINA3-8*. They share approximately 70% of identity with the human *SERPINA3 *homologue. In the cluster, we described an original sub-group of six members with an unexpected high degree of conservation for the reactive-centre-loop domain, suggesting a similar peptidase inhibitory pattern. Preliminary expression analyses of these bovSERPINA3s showed different tissue-specific patterns and diverse states of glycosylation and phosphorylation. Finally, in the context of phylogenetic analyses, we improved our knowledge on mammalian SERPINAs evolution.

**Conclusion:**

Our experimental results update data of the bovine genome sequencing, substantially increase the bovSERPINA3 sub-family and enrich the phylogenetic tree of serpins. We provide new opportunities for future investigations to approach the biological functions of this unusual subset of serine proteinase inhibitors.

## Background

Serine peptidase inhibitors represent a constantly expanding group of structurally related proteins. Amongst them, the most important family is undoubtedly the serpins, an acronym for ***ser***ine ***p***roteinase ***in***hibitors [1] which describes the functional properties of the superfamily. The name serpin was originally coined in recognition of the fact that most serpins are inhibitors of serine proteinases. However, it is now clearly inappropriate because few members of this superfamily lacked any proteinase inhibitory properties [2,3]. Approximately 500 serpins have been identified to date and can be found in all superkingdoms including animals, plants, bacteria as well as some viruses [4,5]. Both extracellular and intracellular serpins have been identified [6]. Most of them are glycoproteins (MW 40,000–60,000) composed of a single polypeptide chain and variable number of oligosaccharide moieties [7]. The protein structure of serpins is characterized by 3 β-sheets and 8 or 9 α-helices [8]. Serpins present a conserved domain, the reactive-centre-loop domain (RCL) which connects β-sheets A and C and often acts as "bait" for the target serine protease [9]. Phylogenetic relationships between orthologous and paralogous serpins have been studied. Irving *et al*. [4] compared several hundred serpin proteins and proposed an arbitrary classification into eight major and eight minor sub-families from A (antitrypsin-like) to P (plants), based on clade recognitions and supports. In vertebrates, serpins are involved in many extracellular processes dependent on serine proteinases such as blood coagulation, fibrinolysis, cell migration, complement activation and inflammation [10,9]. Serpin dysfunction can have pathological consequences and contributes to diseases such as thrombosis, cancer [11] and serpinopathies including cirrhosis and emphysema [12]. Serpins are subdivided into the nine first groups A to I. The two largest groups so far, in all of the serpins, are the antitrypsin-like and the ovalbumin-like serpins which are designated "SERPINA" and "SERPINB" respectively and composed of 13 functional members each. In the "SERPINA" clade, some of them are involved in a diversity of biological functions. For example, human SERPINA3 (α_1_-antichymotrypsin) is found and identified as a major component of the fibrillary amyloid plaques of brains from patients with Alzheimer's disease, one of the most common forms of dementia [13,14].

According to the structure of their respective genes (number of introns and exons and relative position at genomic level), the vertebrate serpin superfamily was subdivided into at least six groups [15]. Most known serpin genes, including the α_1_-antichymotrypsin which belongs to group 2, contain a non-coding first exon and a partly non-coding last exon.

Clustering of serpin genes occurs in the genome of human and other species. They present similarities in gene structure: the *SERPINA *genes characteristically consist of four exons with identical positioning and phasing of the intron-exon boundaries. *SERPINA1 *(α_1_-antitrypsin), *SERPINA3 *(α_1_-antichymotrypsin), *SERPINA5 *(PCI, protein C inhibitor), *SERPINA9 *(centerin), *SERPINA10 *(ZPI, protein Z-dependent protease inhibitor) as well as *SERPINA11 *are mapped together in the same cluster on human chromosome 14q32 [16]. More recently, the gene encoding the SERPINA4 (kallistatin precursor) was also mapped within this cluster. This close proximity in the same cluster suggests that these genes might have arisen by tandem duplications from a common gene ancestor [16,17]. However, in spite of their close physical proximity, the corresponding proteins have disparate functions, raising interesting questions about the evolution of gene function and expression. For example, the SERPINA9 is likely to function *in vivo *in the germinal centre B-cells [18]; the SERPINA5, in addition to its activities within the blood clotting and fibrinolytic cascades, seems to participate in several biological processes including reproduction and tumor growth [19] and SERPINA10 produces a rapid inhibition of the coagulation factor Xa in the presence of protein Z (PZ), procoagulant phospholipids and Ca^2+ ^[20]. An approximate protein similarity of 40–45% between the human SERPINAs [21] could explain these diverse physiological functions.

In mouse, the genomic organization of the serpin family shows significant differences. The cluster of genes corresponding to human chromosome 14q32 is mapped on the chromosome 12F1 [22]. Both α_1_-protease inhibitor and contrapsin genes had undergone considerable expansion in this genome. The mouse α_1_-protease inhibitor (α_1_-antitrypsin – SERPINA1) cluster comprises 5 members. The multigenic cluster corresponding to contrapsin (α_1_-antichymotrypsin – SERPINA3) is termed the *Spi-2 *locus [23] and, until recently, was thought to encompass 14 members [24] with a degree of overall sequence similarity of 65 to 80%, despite markedly divergent RCL domains. Some of these genes present a significant degree of sequence homology with the single-copy human *SERPINA3 *gene [25]. However, the RCL was substantially different. In mouse genome, these two clusters represent the result of multiple gene duplication events.

In the pig, four serine protease inhibitors were detected at the protein level: PI1 (SERPINA1), PI2 (SERPINA3-1), PI3 and PI4 (SERPINA3 paralogues) [26-28]. PI1 and PI2 corresponding genes were mapped by radioactive in situ hybridization (RISH) to the distal end of chromosome 7q23-26 [29,30]. A complete cDNA, named *SERPINA3-1*, corresponding to PI2 and a gene, named *SERPINA3-2*, were sequenced [31] and clearly affiliated to the porcine α_1_-antichymotrypsin (SERPINA3) family. Porcine PI2 and SERPINA3-2 only showed 76% amino acid identity, with the largest difference near the C-terminus containing the deduced reactive site P1-P'1.

The knowledge on bovine serpin genes is much more restricted. Hwang *et al*. [32] identified a serpin, previously named Endopin1, at the cDNA level, in bovine endocrine chromaffin cells. The same serpin was then purified from bovine skeletal muscle [33]. This serpin (mEndopin1A) was found to be essentially intracellular and widely distributed in bovine tissues suggesting that this protein might have central biological role in a large set of tissues [34]. More recently, a second endopin, named mEndopin1B, was purified from bovine diaphragm and the genes encoding mEndopin1A and mEndopin1B were characterized [35].

In order to clarify the complexity surrounding the bovine *SERPINA3 *genes, we describe here the isolation, the complete sequence and the genomic organization of the bovine α_1_-antichymotrypsin (SERPINA3) multigenic cluster. This cluster was assigned to chromosome 21 using a radiation hybrid panel. In addition, we provide preliminary expression data on the bovine *SERPINA3 *genes. Characterization of these new genes and phylogenetic analysis of the corresponding proteins provide new insights on the evolution of *SERPINA3 *gene cluster. We also propose a nomenclature to designate all the members of this clade.

## Results

### Southern blot analyses of *SERPINA3 *genes

**Figure 1:**
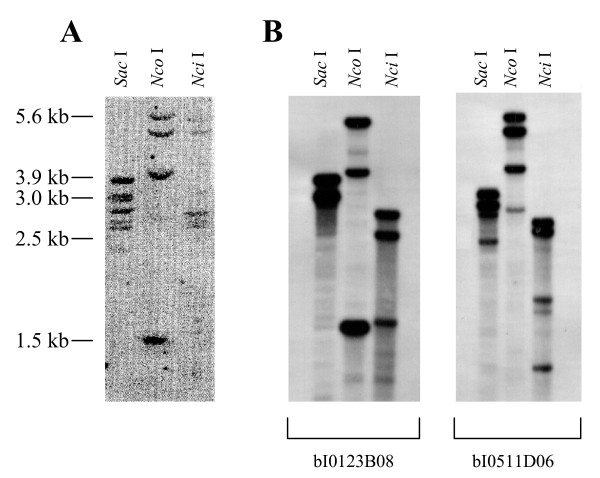
**Southern blot analyses of bovine *SERPINA3 *organization**. **(A) **Southern blot analysis of bovine genomic DNA digested with three different restriction enzymes *Nco*I, *Nci*I and *Sac*I. **(B) **Southern blot analysis of DNA from two overlapping BAC clones bI0123B08 and bI0511D06 digested with the same restriction endonucleases.

Genomic DNA extracted from bovine blood cells was digested to completion with *Nci*I, *Nco*I and *Sac*I endonucleases. After transfer on a Hybond-N^+ ^membrane, DNA was hybridized with a radiolabelled probe corresponding to a part of exon 2 of mEndopin 1A gene [GenBank: AY911536]. After washing under high stringency conditions, several fragments of variable intensity were detected for each endonuclease digest as showed in Figure 1A. A minimum of four hybridizing bands was observed for each DNA digestion. In particular, five bands with variable intensity were obtained using *Sac*I. Several fragments were also observed with an additional experiment performed on isolated BAC DNA (see below) treated in the same conditions (Figure 1B). These Southern blot patterns are consistent with the existence of more than the two genes *mEndopin1A *and *mEndopin1B *previously described [35]. It suggests a complex organization in the bovine genome with more than a round of duplication during evolution from one ancestral *SERPINA3 *gene.

### Molecular cloning of bovine *SERPINA3 *genes

A bovine genomic BAC library was screened by PCR with the set of primers described in Methods section. Four positive BAC clones, named bI0123B08, bI0511D06, bI0311E07 and bI0382C01, were identified. Only bI0123B08 and bI0511D06 BAC DNAs were then subjected to long-range PCR with two sets of primers (see methods).

After cloning of the PCR products, several positive clones with inserts ranging from 7.0 to 8.0 Kb were identified with a first set of primers corresponding to the 5' and 3' ends of the *mEndopin1A *cDNA sequence. Restriction mapping using different endonucleases revealed six different profiles. Two of them were first analyzed and assigned to mEndopin1A and mEndopin1B proteins, which were already found and purified from bovine skeletal muscle [35]. In our new proposal of nomenclature, the corresponding genes are named *SERPINA3-1 *[GenBank: AY911536] and *SERPINA3-3 *[GenBank: AY911537], respectively. The four other fragments were also analyzed, sequenced on both strands using several sense and anti-sense primers as previously described [35] and assigned to four new genes: *SERPINA3-2 *[GenBank: EF153626], *SERPINA3-4 *[GenBank: EF153627], *SERPINA3-5 *[GenBank: EF153628] and *SERPINA3-6 *[GenBank: EF153629].

**Figure 2:**
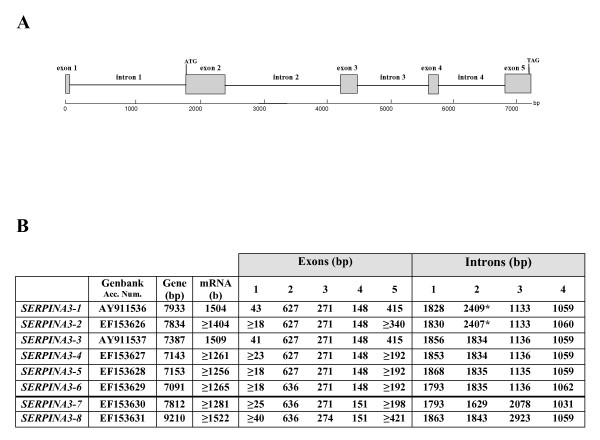
**Genomic organization of bovine *SERPINA3 *genes**. **(A) **General and schematic representation of *SERPINA3 *genes. Exons numbered from exon 1 to exon 5 are indicated by gray boxes, and intron sequences intron 1 to intron 4 by a thin line. **(B) **The Genbank accession numbers, the size in bases of each gene, mRNAs, exons and introns are listed in the table. The horizontal line in bold type separates the sub-group of the two other genes. The asterisk in intron 2 of *SERPINA3-1 *and *SERPINA3-2 *genes indicates the presence of the Bov-B LINE sequence.

Three clones with different restriction profiles were obtained with a second set of primers corresponding to the 5' end of the *SERPINA3 *EST [GenBank: DT861683] and the 3' end of the *endopin2B *cDNA sequence [GenBank: AY386699] published by Hwang and collaborators [36]. The clones were sequenced on both strands and assigned to *SERPINA3-7 *[GenBank: EF153630], *SERPINA3-8 *[GenBank: EF153631] and *SERPINA3P *[GenBank: EF153625]. Comparison of these genomic DNA sequences, using the online software Spidey [37], with the *SERPINA3-1 *cDNA allowed us to deduce the gene structures. Sizes of exons and introns, as well as exon/intron junctions, were determined. Genes are split into five exons (exon 1 to exon 5) and four introns (intron 1 to intron 4), as presented in Figure 2A. Figure 2B shows the sizes of exons and introns for the first eight bovine *SERPINA3 *genes. These genes have a good conservation of the introns and exons size, except for the intron 2 of *SERPINA3-1 *and *SERPINA3-2*, which is due to the insertion of a Bov-B LINE. Finally, for all these genes, the translation initiation codon ATG is located in exon 2, few base pairs downstream intron 1/exon 2 boundary and the stop codon TAG is found in exon 5. Due to the conserved gene organization of class 2 serpins (five exons and four introns), a potential pseudogene (*SERPINA3P*) was also identified. Compared to the other *SERPINA3 *cDNAs, *SERPINA3P *gene is partially truncated at the end of the exon 3 and the beginning of the exon 4. In addition a 13 bp deletion in exon 2 induces a frame shift which generates a premature stop codon TGA at position 2289 in exon 2. Figure 2B indicates also the size of isolated mRNAs for *SERPINA3-1 *and *SERPINA3-3*, and of deduced mRNAs for *SERPINA3-2, SERPINA3-4, SERPINA3-5, SERPINA3-6, SERPINA3-7 *and *SERPINA3-8*.

### Localization and genomic organization of bovine *SERPINA3 *genes

**Figure 3:**
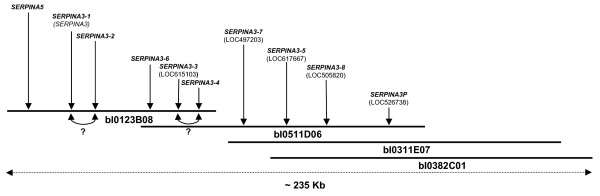
**Structure of the *SERPINA3 *locus on *Bos taurus *chromosome 21**. The genes are labelled according to the classification in Figure 2B. Their names are indicated in bold characters and the names of loci established for Btau-2.0 genomic sequence assembly are added (when known) below between brackets. The different genes are localized on the four overlapping BAC clones covering approximately 235 Kb. The two question marks indicate the uncertainties of relative location for the genes *SERPINA3-1*/*SERPINA3-2 *and *SERPINA3-3*/*SERPINA3-4*, respectively. The *SERPINA5 *gene is also shown.

*SERPINA3 *genes localization within the bovine genome was performed using a radiation hybrid (RH) panel. The Roslin 3000-rad RH panel [38] was screened by PCR using primers able to amplify a specific DNA fragment of 477 bp in exon 2. Results revealed a single localization on bovine chromosome 21 between *ILSTS054 *(635 cR) and *IDVGA39 *(658 cR) markers. The use of a first generation bovine BAC-based physical map [39] showed that the four BAC clones previously isolated were contained in the single BAC contig 2018 [40] and covered approximately 235 Kb (Figure 3). Using the publicly available 6× bovine genomic sequence assembly (Btau_2.0) [41], we confirmed the RH localization and assigned the bovine *SERPINA3 *genes to the region 21q24. Comparisons of DNA sequences allocate some of our sequenced genes to predicted genes in Btau_2.0, like *SERPINA3-1 *to *SERPINA3*, *SERPINA3-3 *to LOC615103, *SERPINA3-5 *to LOC617667, *SERPINA3-7 *to LOC497203, *SERPINA3-8 *to LOC505820 and *SERPINA3P *to LOC526738. The genomic organization of the nine bovine *SERPINA3 *genes was deduced from these results and the BAC Southern blot analysis (Figure 3). Allocated genes are localized on two distinct sequence contigs in bovine build 2.0 genome (NW_930022 and NW_930023) and covered up to 1.5 Mb. The relative positions of *SERPINA3-1*/*SERPINA3-2 *and *SERPINA3-3*/*SERPINA3-4 *were not yet elucidated. Moreover, we only succeeded in localizing the *SERPINA5 *gene upstream *SERPINA3-1 *in bI0123B08 BAC clone using the MARC_10319-10320 STS [UniSTS: 267110].

### Analysis of deduced SERPINA3 protein sequences

**Figure 4:**
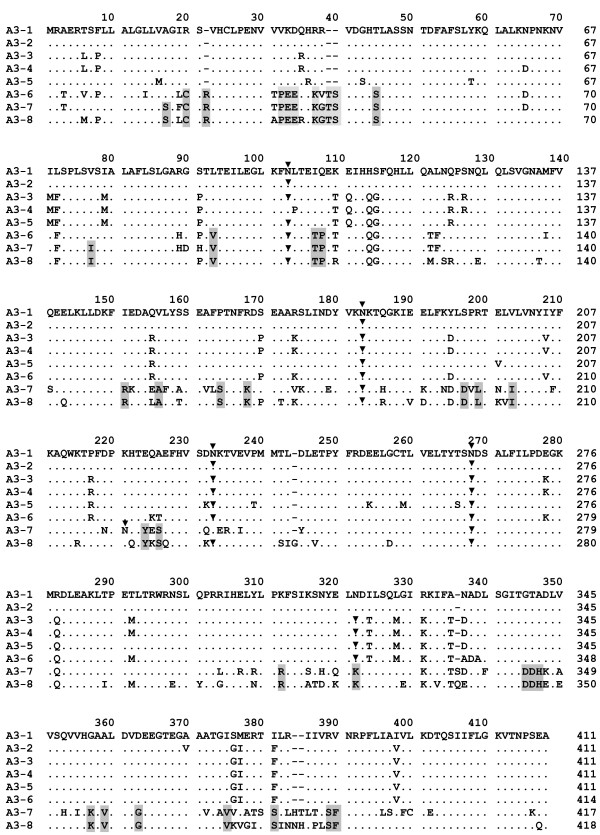
**Multiple amino acid sequence alignment of the bovine SERPINA3 family**. The eight putative bovSERPINA3 proteins were aligned using CLUSTALW [60]. Numberings refer to position in the alignment, above the sequences, and to the length of each sequence, at the end of the line. Dot indicates a common residue with that of the first sequence (bovSERPINA3-1), dash shows a gap, i.e. a position with an insertion-deletion event. Shaded characters indicate selectively conserved residues specific of the sub-group including bovSERPINA3-7 and bovSERPINA3-8. (▼) indicates asparagine (N) residue involved in a potential N-glycosylation site.

The deduced amino-acid primary sequences of these eight bovine *SERPINA3 *genes indicated that protein sizes range from 411 to 418 amino acids. Different numbers of potential N-glycosylation sites (N-X-S/T with X neither P) were present (Figure 4). The bovSERPINs A3-1/A3-2/A3-3/A3-4 and A3-5 have very similar sequences with 95% of identity and separate from bovSERPINA3-7 and A3-8, only sharing 74% of identity with them. Based on protein alignment, a sub-group of bovSERPINA3 could be distinguished. It is composed of bovSERPINA3-1, bovSERPINA3-2, bovSERPINA3-3, bovSERPINA3-4 and bovSERPINA3-5. The membership of the bovSERPINA3-6 is much more ambiguous. As seen in Figure 4, this protein shares more resemblance with that sub-group in its C-terminal part, but its N-terminal part is closer to bovSERPINA3-7 and bovSERPINA3-8.

### Preliminary RT-PCR analysis of bovine *SERPINA3 *genes

RT-PCR was used to determine the presence of *SERPINA3 *mRNAs in bovine skeletal muscles (*semitendinous *and *diaphragma*) and also in liver and kidney. Because of the high homology between the eight *SERPINA3 *genes only three primer pairs were used (see methods).

RT-PCR, with the primer set SERPINA3-1/F, SERPINA3-1/R, amplified 319 bp fragments in all tested tissues (data not shown). Two different sequences were obtained. The first sequence is present in all tested tissues and would correspond to *SERPINA3-1 *and/or *SERPINA3-2 *transcripts, which could not be discriminated by this portion of sequence. The second sequence is only present in the liver. It would correspond to *SERPINA3-3*, *SERPINA3-4 *and/or *SERPINA3-6 *transcripts, which could not be discriminated by this portion of sequence.

Primers SERPINA3-6/F, SERPINA3-6/R and primers SERPINA3-5/F, SERPINA3-5/R allowed to amplify a DNA fragment only in the liver. Surprisingly, with the primer pair SERPINA3-6/F, SERPINA3-6/R, after sub-cloning, amplifying and sequencing, a single sequence corresponding to the *SERPINA3-7 *mRNA was obtained. This RT-PCR was supposed to generate a specific amplification of *SERPINA3-6 *transcript. However, if *SERPINA3-6 *cDNA is one of the minor isoforms of bovine *SERPINA3 *in liver, it may not be detected by RT-PCR. With the primers SERPINA3-5/F and SERPINA3-5/R, several sequences were obtained. They are related to *SERPINA3-3*, *SERPINA3-4 *and *SERPINA3-5 *cDNAs but are different from the other cDNAs of the *SERPINA3 *family.

### Proteomic analysis of muscular bovine SERPINA3

**Figure 5:**
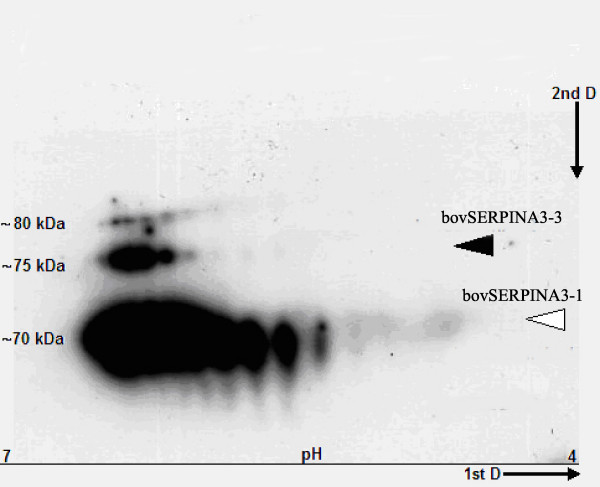
**2D gel Western blot of a bovine SERPINA3 enriched fraction**. High Mr fraction is eluted from a bovine *diaphragma *crude extract after gel filtration on a Sephadex G100 column, separated on 2D gel electrophoresis and hybridized with specific polyclonal antibodies. Arrows indicate the position of the two first characterized serpins bovSERPINA3-1 and bovSERPINA3-3. Note the linear alignment of spots corresponding to a different extent of phosphorylation of isoforms running with similar Mr on the second dimension. For more detail see the Methods section.

According to the Western blot carried out after 2D-gel electrophoresis of the Sephadex G100 F1 fraction (Figure 5), the antibody revealed series of spots with at least three different molecular weights and ranging from 70 to 80 KDa. The calculated relative mass (Mr) of the eight deduced proteins is 46 KDa. The difference of electrophoretic profile could be explained by different levels of glycosylation. Each initial spot with different Mr spreads out on the pH gradient indicating a large variation in the extent of phosphorylations of different isoforms. Their pI values vary from 6 to 4.5. For each Mr, the spots probably correspond to a set of bovSERPINA3 isoforms which are not resolved in the present conditions. For information, the position of the two purified isoforms including bovSERPINA3-1 (70 KDa) and bovSERPINA3-3 (75 KDa) are indicated in Figure 5. The present results clearly demonstrate that a relatively large set of bovSERPINA3 are expressed in skeletal muscle.

### Phylogenetic analyses of SERPINAs

**Figure 6:**
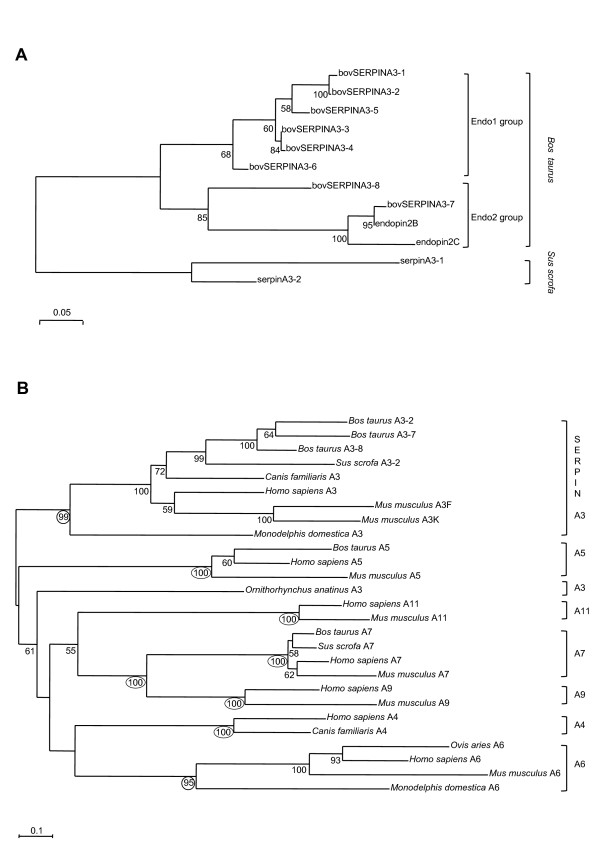
**Phylogenetic trees including bovSERPINA3s**. **(A) **BovSERPINA3 phylogeny based on Maximum likelihood (ML) method. The best ML tree (LnL = -3648.09) is rooted on porcine SERPINA3s. **(B) **SERPINA phylogeny based on ML method with the closest relatives of SERPINA3. The best ML tree (LnL = -16085.84) is unrooted. Bootstrap proportions are given for nodes supported in at least 50% cases. BP supporting clades are circled.

Analyses were performed on the deduced amino acid sequences. BovSERPINA3 phylogeny based on maximum likelihood (Figure 6A) illustrates a complex situation among this sub-family. Three pairs of sequences (A3-1/A3-2, A3-3/A3-4 and A3-7/endopin2B) are recognized with high bootstrap proportions (BPs), ranging from 84% to 100%. They would be the result of very recent gene duplications in the *Bos taurus *genome, which would be supported by their proximity on the chromosome 21. A dichotomy is seen, which separates the endo1 group (named according to endopin1) including bovSERPINA3-1 to A3-6, from the endo2 group (named from endopin2) including bovSERPINA3-7, A3-8, endopin2B and 2C. Relative orders of emerging within endo1 group are not resolved (BPs ranging from 58% to 68%), but bovSERPINA3-6 is very likely to be part of this group even if the N-terminal part of its amino acid sequence showed more similarities with bovSERPINA3-7 and A3-8. Finally, the degree of sequence divergence within the endo1 group is lower than in the endo2 one.

SERPINA3 amino acid sequences were also analysed in the context of a larger phylogeny (Figure 6B) including the closest SERPINA families based on BLAST [42] similarities and recognized as a part of its sub-group 2 by van Gent *et al*. [21]. All clades of SERPINA used in this study are supported by high bootstrap values (95 to 100%), except for SERPINA3 clade due to the highly divergent sequence of *Ornithorhynchus anatinus *SERPINA3. Phylogenetic relationships between all clades remain unsolved. However, one exception with SERPINA7 (thyroxine-binding globulin) and SERPINA9 (centerin) which are clearly sister-groups with BP of 100%. In addition to SERPINA3, SERPINA5 [43] and SERPINA7 [44] are found in the *Bos taurus *genome. However, only for SERPINA3 several genes are found especially in mouse.

## Discussion

Two bovine serpins named mEndopin1A (renamed here bovSERPINA3-1) and mEndopin1B (renamed here bovSERPINA3-3) were recently purified from *diaphragma *and characterized [33,35]. Although the sequence identity between both muscle endopins is close to 93%, the major differences were found within the RCL. These two proteins are homologous to the unique human SERPINA3 and share with it a high identity (approximately 67%). The genes encoding these two proteins were shown to be expressed notably in muscle. In this study, we show that bovSERPINA3-1 and bovSERPINA3-3 are members of a family comprising at least six other deduced proteins. The genes encoding these proteins are mapped to the presently named *SERPINA3 *cluster localized on chromosome 21q24. Our results indicate that the locus spans over ~235 Kb and contains at least eight *SERPINA3 *genes with a local order of *SERPINA3-1*/*SERPINA3-2*, *SERPINA3-6*, *SERPINA3-3*/*SERPINA3-4*, *SERPINA3-7*, *SERPINA3-5 *and *SERPINA3-8*, and the pseudogene *SERPINA3P *localized at the 3'end of the cluster. We also mapped another *SERPINA *gene, *SERPINA5 *at the 5' end of *SERPINA3-1/SERPINA3-2*. This mapping is not in agreement with the publicly available Btau_2.0 genomic sequence assembly. To correlate with our proposed genomic organization based on the physical map [39], it was necessary to invert the relative positions of the two contigs (NW_930022 and NW_930023) on chromosome 21. However, all the genes identified in this work had not been listed in Btau 2.0 assembly. To date, the *SERPINA3-2*, *SERPINA3-4 *and *SERPINA3-6 *genes are not identified in the whole genome shotgun sequence data. This absence is probably caused by the high degree of similarity between bovine *SERPINA3 *genes. The current Btau 3.1 [41] doesn't clarify the situation. Indeed, the allocated genes are still the same but are now localized on two contigs distant of 2 Mb from each other (NW_001494061 and NW_001494063). The new Btau 4 version, not yet publicly available, might help us to resolve this discrepancy.

All these genes share an exon-intron organization identical to that of class 2 serpin genes, as established by Ragg and collaborators [15] with four coding exons and one untranslated exon. Moreover, they share a high degree of identity both in exonic and intronic sequences. Therefore, all these genes established a compact bovine *SERPINA3 *family with at least eight functional members and one pseudogene. *SERPINA3 *gene clusters exist in other mammal genomes. *Mus musculus *genome (build 37.1 [45]) contains nine expressed *SERPINA3 *genes and two pseudogenes located on chromosome 12. The deduced proteins display only 55% to 80% of identity. Porcine *SERPINA3 *sequence analysis defined at least three expressed genes [UniGene:55446, UniGene:50917, UniGene:3917] [31] with comparable degree of overall identity as observed in mice. On the other hand, primate genomes only possess a single representative *SERPINA3 *gene, as seen in rhesus macaque, chimpanzee and human, the latter being located on chromosome 14q32 [16].

We described, for the first time in mammals, a serpin sub-family of eight genes with one sub-group composed of six very conserved members: *SERPINA3-1*,*SERPINA3-2*,*SERPINA3-3*,*SERPINA3-4*,*SERPINA3-5 *and *SERPINA3-6*, with high degree of identity over the full sequences (from 88 to 99%). Members of the bovine *SERPINA3 *sub-family arose by recent duplications after divergence from *Sus scrofa *and from an ancestral gene, located on the region 21q24, homologous to the unique primate *SERPINA3 *gene. They show a considerable gene clustering as the mouse ones, but with notable differences. Indeed, rodent *SERPINA3 *homologues are grouped into 4 sub-clades due to three rounds of duplication prior to speciation between mouse and rat. Following duplications, mainly in mouse, were then lineage-specific [46]. All these *SERPINA3 *cluster organizations are analogous to the one observed at the family level, that is for *SERPINA*. In case of human, where 13 members are described, only *SERPINA7 *and *SERPINA8 *are located on different chromosomes, Xq22 and 1q42-43 respectively, whereas all others are on chromosome 14q32. They probably also arose by several rounds of duplication within as well as between chromosomes as already demonstrated for human ovalbumine-like serpins (SERPINB) [47]. The single clade supported by 100% BP on ML tree clustered SERPINA7 and SERPINA9, although these most related members are on distinct chromosomes and present different activities, non-inhibitory and inhibitory respectively. This suggests that through evolution, the loss of inhibitory function in some SERPINA occurred several times independently, as seen for SERPINA7 but also for SERPINA6. Finally, in bovine, although the genome is completely sequenced, only four sub-families were identified to date, with *SERPINA1*, *SERPINA3 *and *SERPINA5 *assigned to chromosome 21 and SERPINA7 probably on chromosome X as all other mammal *SERPINA7*s.

The potential active domain of serpins, in the C-terminus loop, contains the reactive site P1-P'1 responsible for the serpin specificity. In the sub-family of bovSERPINA3 described in this paper, the RCL (Table 1) has been identified by analogy with other members of the superfamily of serpins. As indicated in this table, boxed residues in bold characters are strongly conserved between these eight putative proteins and are specific of inhibitory serpins [48]. Moreover, for the sub-family including bovSERPINA3-1 to bovSERPINA3-6, the full RCL sequence is remarkably conserved with more than 82% of identity. This high degree of conservation was not found in mice and porcine SERPINA3s. We suggest that these six bovine serpins might have a similar peptidase inhibitory pattern. Indeed, we were already able to establish for the two serpins, bovSERPINA3-1 and bovSERPINA3-3, purified and characterized from bovine *diaphragma *muscle, a similar peptidase inhibitory pattern against the same proteases, elastase and trypsin [35]. These six serpins could represent an original sub-family of the SERPINA3s in bovine and not found so far in other mammals.

**Table 1:** Comparison of RCL sequences for bovSERPINA3s

**bovSERPINA3-1**	361	**G**	**T**	**E**	**G**	**A**	**A**	**A**	**T**	G	I	S	M	E	R	T	I	L	R	-	-	I	I	V	R	382
**bovSERPINA3-2**	361	·	·	·	·	**V**	·	·	·	·	·	G	I	·	·	·	F	·	·	-	-	·	·	·	·	382
**bovSERPINA3-3**	361	·	·	·	·	·	·	·	·	·	·	G	I	·	·	·	F	·	·	-	-	·	·	·	·	382
**bovSERPINA3-4**	361	·	·	·	·	·	·	·	·	·	·	G	I	·	·	·	F	·	·	-	-	·	·	·	·	382
**bovSERPINA3-5**	361	·	·	·	·	·	·	·	·	·	·	G	I	·	·	·	F	·	·	-	-	·	·	·	·	382
**bovSERPINA3-6**	364	·	·	·	·	·	·	·	·	·	·	G	I	·	·	·	F	·	·	-	-	·	·	·	·	385
**bovSERPINA3-7**	365	·	·	·	·	·	·	**V**	·	A	V	V	·	A	T	S	S	·	L	H	T	L	T	·	S	388
**Endopin 2B**	365	·	·	·	·	·	·	**V**	·	A	V	V	·	A	T	S	S	·	L	H	T	L	T	·	S	388
**bovSERPINA3-8**	366	·	·	·	·	·	·	·	·	·	V	K	V	G	I	T	S	I	N	N	H	·	P	L	S	389
**Endopin 2C**	362	·	·	·	·	·	·	**V**	·	A	V	I	·	F	T	S	L	P	L	H	A	L	N	I	S	385

The potential RCL within the C-terminus loop is responsible for the specificity of inhibition. It was identified for the eight putative bovSERPINA3s by analogy with other serpins. Boxed residues in bold characters appeared to be more specific of inhibitory serpins [48]. The boxed arginine residue (R) for bovSERPINA3-1 to bovSERPINA3-6 could be the P1 residue for trypsin cleavage [33, 35]. The boxed threonine residue (T) for endopin2B could be the primarily P1 residue for elastase cleavage [49]. Numberings at the two extremities refer to positions in each full sequence. Dots indicate identical residues compared to those of bovSERPINA3-1 and dashes show gaps in the alignment.

SERPINA3-7 corresponds to the previously identified endopins 2A/2B [49,50]. However, despite using specific set of primers, we could not amplify the third bovine endopin 2, named 2C [36] on our four different BAC DNAs.

In mice and porcine, no specific expression analyses have been conducted on proteins encoded by *SERPINA3 *genes of clusters. However, although bovSERPINA3s are very close, they share differential expression patterns. For example, *SERPINA3-1 *is expressed in all tested tissues, whereas *SERPINA3-4 *is amplified only in liver. Moreover, consensus glycosylation sites and the 2D-gel analysis predicted that bovSERPINA3 may be glycoproteins with different degrees of N-glycosylation. In a previous study [35], we have already shown that four potential N-glycosylation sites (Asn^100,180,230,264^) are common to bovSERPINA3-1 and bovSERPINA3-3. However, an additional site (Asn^318^) is present in bovSERPINA3-3 and could account for the slightly higher molecular mass (75 KDa for bovSERPINA3-3 *versus *70 KDa for bovSERPINA3-1) as assessed by SDS-PAGE. So, as indicated in the 2D-gel Western blot analysis of a partially purified muscle bovSERPINA3 fraction (Figure 5), several bovSERPINA3s present various states of phophorylation. Protein phosphorylation is a very important signalling pathway since this post-translational modification ensured by a large set of specific Ser/Thr/Tyr kinases, might be essential for the biological activity of the protein concerned and/or for a modulation of this activity [51,52]. Those observations lead us to propose a differential functionality for these protein isoforms.

## Conclusion

Comprehensive analysis of the bovine genome *SERPINA3 *content combined with phylogenetic clade recognition has allowed us to characterize one original *SERPINA3 *gene cluster not identified yet in other mammals. Some of these eight corresponding proteins were shown to be expressed in various tissues and differently glycosylated and/or phosphorylated. This underlines a complex mechanism of regulation for the biological function of bovine SERPINA3s.

This coherent sub-family of serpins could constitute a model to study the duplication events and their underlying molecular proceedings at the origin of multigenic families.

Actually, it is interesting to notice that the numerous SERPINA3s of *Bos taurus *contrast with the paucity of other SERPINA sub-families compared to *Homo sapiens *(5 assigned and 3 predicted in bovine [41] versus 13 in human).

## Methods

### Isolation of genomic BACs encoding bovine *SERPINA3 *genes

A bovine genomic BAC Library [53] was screened at the Centre de Ressources Biologiques (INRA, Jouy-en-Josas, France) by PCR for the isolation of the *SERPINA3 *genes. PCR was performed in 50 μl volume reaction using the primers 5'-TGAGGGCAGAGAGAACTTCCTTC-3' and 5'-AGGCCTCAGAGGAGTACAGCAC-3', designed with reference to the cDNA sequence of bovine chromaffin cell Endopin 1 [GenBank: AF125526], 1.5 mM MgCl_2_, 2.5 Units *Taq *DNA polymerase (Invitrogen, Carlsbad, USA) and thermocycling consisting of 94°C for 3 min followed by 94°C for 30 s, 55°C for 30 s and 72°C for 45 sec for 35 cycles. DNA from the positive BACs was isolated using a Qiagen^®^Large-Construct Kit tip 100 column (Qiagen Inc., Hilden, Germany) according to the manufacturer's recommendations.

### Subcloning and DNA sequencing

To identify intron/exon boundaries and to provide sequence of introns and exons of each gene, BAC DNA was subjected to long-range PCR with the two following primers pairs: 5'-TGGATCCACTGCCCACATCCCGCTC-3' and 5'-CTAGGCTTCACTGGGGTTGGTGAC-3' designed with reference to Bos taurus chromosome 21, reference assembly (based on Btau_3.1), whole genome shotgun sequence [GenBank: NC_007319] c52366530-52358602; 5'-AGCATCCGGGCAGGCGGCGGCTCTGGATCCACTGC-3' and 5'-CTAGGCTTCCTTGGGGTTGGTGACTTTGCCC-3' designed with reference to Bos taurus chromosome 21, reference assembly (based on Btau_3.1), whole genome shotgun sequence [GenBank: NC_007319] 54262873–54270895. The first set of primers was able to amplify *SERPINA3-1 *to *SERPINA3-6 *genes and the second *SERPINA3-7 *and *SERPINA3-8 *genes, using the Expand Long Template PCR System (Roche Molecular Biochemicals, Mannheim, Germany). The 50 μl PCR reaction contained 15 pmol of each primer, 2.5 units of DNA polymerase, 25 nmol of each dNTP, 50 ng of DNA and 2.25 mM MgCl_2_. Amplifications were performed using the following cycling parameters: one cycle of denaturation at 94°C for 2 min, followed by 10 cycles of 10 s at 94°C, 30 s at 61°C, 15 min at 68°C, 20 cycles of 10 s at 94°C, 30 s at 61°C, 15 min at 68°C, incremented by 20 s at each cycle, followed by a final elongation at 68°C for 10 min. Amplification products were cloned using the pCR-XL-TOPO vector (Invitrogen) before sequencing. The complete sequences were obtained on both strands using several primers as described previously [35] and genomic organization of bovine *SERPINA3 *genes was determined by alignments using the Sequencher™ 4.1.4 software (GeneCodes Corp., Ann Arbor, USA).

### Southern blot analysis

Bovine genomic DNA was prepared from blood samples using the QIAmp Blood kit (Qiagen Inc.). Ten micrograms of bovine genomic DNA and screened BAC DNA were subjected to digestion with *Sac*I, *Nco*I and *Nci*I restriction endonucleases. Fragments were separated through 0.8% (w/v) agarose gel. DNA was depurinated for 20 min with 0.25 N HCl, denaturated for 30 min with 0.4 N NaOH, and transferred onto a Hybond-N+ membrane (GE Healthcare, Chalfont St. Giles, UK). A 232-bp probe was generated by PCR using 5'-TGAGGGCAGAGAGAACTTCCTTC-3' and 5'-AGGCTATGGAGACACTCAGCG-3' as primers and bovine *mEndopin1A *(*SERPINA3-1*) cDNA as probe. Twenty-five nanograms were labelled with [^32^P]dCTP by random priming (Random Primers DNA labeling System, Invitrogen), purified to avoid unincorporated isotope (QIAquick nucleotide removal kit, Qiagen Inc.) and was used with a specific activity of 5.10^8 ^cpm/mg. Hybridizations were carried out for 12 h at 65°C in a buffer containing 10% (w/v) dextran sulfate, 1% (w/v) SDS, 0.5 M NaCl, and 100 μg of sheared salmon sperm DNA. Blots were washed three times at 42°C for 10 min each with 2× SSC; 2× SSC 0.1% SDS and 1× SSC 0.1% SDS and then analyzed by PhosphorImager (Molecular Dynamics, Sunnyvale, USA).

### Chromosomal localization of bovine *SERPINA3 *genes

The localization of *SERPINA3 *genes was performed using the Roslin 3000-rad RH panel [38]. The bovine genes were typed on DNA from the 94 radiation hybrid lines together with control bovine and hamster DNA by PCR in 96-well microtitre plate using the set of primers 5'-TGAGGGCAGAGAGAACTTCCTTC-3' and 5'-AGGCCTCAGAGGAGTACAGCAC-3' able to amplify a specific DNA fragment of 477 bp of exon 2. PCR reactions were performed in 20 μl with 25 ng DNA, 1 pmol of each primer, 50 mM KCl, 10 mM Tris-HCl pH 9.0, 1 μM dNTPs, 0.5 unit of Upti Therm DNA polymerase (Interchim, Montluçon, France) and 1 mM MgCl_2_. The PCR was started with 3 min at 94°C followed by 35 cycles of 30 s at 94°C, 30 s at 55°C, and 45 s at 72°C for 1 min, with a final incubation at 72°C for 5 min. Reactions were carried out in duplicate. Presence or absence of the 477 bp PCR product in reactions was determined by 96 well mini-agarose gel electrophoresis. PCR fragments were visualized by ethidium bromide stained agarose gels. *SERPINA3 *genes were assigned to chromosome by analyzing 2-point linkage with mapped loci using RH mapper [54].

### RNA isolation and cDNA analysis

Total RNA was isolated from several frozen bovine tissues obtained within 1 h post-exsanguination from the local slaughter house of the INRA Theix Research Centre. Frozen tissue (300 mg aliquots) was pulverized with a polytron (ULTRA-TURRAX^® ^T25 basic IKA^®^-WERKE), solubilized in 1 ml of TRIZOL reagent (Invitrogen), extracted with 0.2 ml chloroform, isoamylalcohol (49/1, v/v) and incubated at room temperature for 5 min. The sample was then centrifuged (12,000 g, 4°C, 15 min) and the resultant RNA present in the aqueous phase was precipitated by isopropanol and resuspended in 50 μl H_2_O.

Reverse Transcription was performed from 1 μg total RNA, in a total volume of 20 μl, using 0.5 μg oligo(dT) primer (Invitrogen) and 5 units of SuperScript™ II RNase H^- ^Reverse Transcriptase (Invitrogen) according to manufacturer's instructions. The reaction was incubated for 50 min at 42°C and 15 min at 72°C. cDNAs were stored at -20°C until use.

First strand cDNA synthesis was followed by PCR conducted with 0.4 mM sense and antisense primers, 1 unit of Upti Therm DNA polymerase (Interchim) and thermocycling consisting of 1 cycle of 3 min at 94°C followed by 35 cycles of 30 s at 94°C, 1 min at 55°C, and 1 min at 72°C, with a final incubation at 72°C for 10 min. Primers SERPINA3-1/F 5'-CCCATGATGACCCTTGACCT-3' and SERPINA3-1/R 5'-TACCAGGTCCGCGGTCCCTGTGAT-3' complementary to *SERPINA3-1 *up to *SERPINA3-6 *were designed to amplify a 319 bp DNA fragment that includes a part of exon 3 and exon 4. Analogously, primers SERPINA3-6/F 5'-ACCCCAGAAGAACAGCACAAAGTGACGTCT-3' and SERPINA3-6/R 5'-CTTGTTGTCGCTCACGTGGAACTCTGTCTTC-3' complementary to *SERPINA3-6 *and primers SERPINA3-5/F 5'-TCCTTCCTCCTGGCCGGGCTCCTGATG-3' and SERPINA3-5/R 5'-GAGGATGAAGAGGGCGCTGTCGTTGCTACT-3' complementary to *SERPINA3-5 *should amplify 615 bp and 797 bp DNA fragments respectively, that includes a part of exon 2 and exon 3. PCR-generated DNA fragments were subcloned into the pEasyT-vector (Promega, Madison, USA) and amplified in TOP10 competent *Escherichia coli *cells. DNA inserts of appropriate size (assessed by digestion of the plasmid with appropriate restriction endonuclease) were subjected to automated DNA sequencing as previously described. For DNA sequencing, we used reverse T7 and forward SP6 primers that flank the DNA insert.

### 2D-gel analysis of a partially purified bovine SERPINA3 fraction

A crude muscle extract first fractionated by differential centrifugation steps was then concentrated by ammonium sulphate precipitation between 40 and 70 % saturation and the pellet suspended in 50 mM Tris/HCl Buffer pH 7.5 containing 5 mM EDTA, 5 mM 2-β mercaptoethanol and dialysed overnight against the same buffer. The dialysed extract was then run on a Sephadex G100 column (100 × 5 cm) at a flow rate of 24 ml. h^-1 ^[35]. The first fraction inhibiting trypsin (F1) was collected and further analysed by 2D gel electrophoresis as previously described [55]. Briefly, about 100 μg of proteins were included in a buffer containing 7 M urea, 2 M thiourea, 2% (w/v) CHAPS, 0.4% (v/v) carrier ampholyte and bromophenol blue. Samples were loaded onto immobilized pH gradient strips (pH 5–8, 17 cm, Bio-Rad, Hercules, USA) and isoelectric focusing was performed using a Protean IEF cell system (Bio-Rad). Gels were passively rehydrated for 16 h. Rapid voltage ramping was subsequently applied to reach a total of 85 kV.h^-1^. In the second dimension, proteins were resolved on 12% SDS-polyacrylamide gel electrophoresis (SDS-PAGE) gels using Protean II XL system (Bio-Rad). Gels were Coomassie Blue (colloidal blue) stained and Western blot was performed.

### Western blot analysis

Anti-bovSERPINA3 antibodies raised against purified bovine mEndopin1 [56] cross-react with all isoforms of bovine serpins identified in this work and named bovSERPINA3s. Separated proteins were then transferred during 40 min at 200 mA onto a nitrocellulose membrane (Schleicher & Schuell, Dassel, Germany). The immunoblot was processed by chemiluminescence detection (Chemiluminescence Blotting Substrate [POD], Roche Molecular Biochemicals). After overnight saturation, the membrane was first incubated with rabbit anti bovSERPINA3 anti-serum (dilution 1:500) for 1 h at room temperature under agitation, and then with a second antibody, a swine anti-rabbit IgG conjugated to horseradish peroxidase (dilution 1:1000) (DAKO, Glostrup, Denmark) for 40 min at room temperature and under agitation.

### Phylogenetic analysis

Details concerning SERPINAs used for phylogenetic reconstructions are shown in Table 2. Sequences were aligned using the program SEAVIEW [57]. Positions including gaps due to the alignment were removed. Twelve sequences and 402 positions, and twenty-seven sequences and 360 positions were retained for phylogenetic analyses of the multigenic sub-family of bovSERPINA3 and their relationship with other SERPINA families, respectively. Maximum likelihood trees were obtained using PHYML version 2.4.4 [58]. We applied the Jones-Taylor-Thornton (JTT) model of amino acid substitution [59], a site-to-site evolutionary rate variation modeled on a Γ distribution with 4 rate categories and the shape parameter estimated from the data. The statistical supports for internal branches were estimated by the non parametric bootstrap method on 500 replicates.

**Table 2:** List of SERPINAs used for the phylogenetic analyses

**SERPINA**	**Organisms**	**Protein names**	**Accession numbers**
**SERPINA3 **(α1-antichymotrypsin precursor)	*Bos taurus*	bovSERPINA3-1	AAY22405
		bovSERPINA3-2	ABM55496
		bovSERPINA3-3	AAY22406
		bovSERPINA3-4	ABM55497
		bovSERPINA3-5	ABM55498
		bovSERPINA3-6	ABM55499
		bovSERPINA3-7	ABM55500
		bovSERPINA3-8	ABM55501
		Endopin2B	AAR26722
		Endopin2C	AAX63906
	*Canis familiaris*		BXP_537546
	*Homo sapiens*		P01011
	*Monodelphis domestica*		XP_001375795
	*Mus musculus*	SERPINA3-F	Q80X76
		SERPINA3-K	P07759
	*Ornithorynchus anatinus*		XP_001516508
	*Sus scrofa*	SERPINA3-1	M29508
		SERPINA3-2	CAC05490
**SERPINA4 **(kallistatin precursor)	*Canis familiaris*		XP_547962
	*Homo sapiens*		P29622
**SERPINA5 **(plasma serine protease inhibitor precursor)	*Bos taurus*		Q9N212
	*Homo sapiens*		P05154
	*Mus musculus*		P070458
**SERPINA6 **(corticosteroid-binding globulin precursor)	*Homo sapiens*		P08185
	*Monodelphis domestica*		XP_001370999
	*Mus musculus*		Q06770
	*Ovis aries*		P49920
**SERPINA7 **(thyroxine-binding globulin precursor)	*Bos taurus*		Q9TT36
	*Homo sapiens*		P05543
	*Mus musculus*		P16939
	*Sus scrofa*		Q9TT35
**SERPINA9 **(centerin precursor)	*Homo sapiens*		Q86WD7
	*Mus musculus*		Q9D7D2
**SERPINA11**	*Homo sapiens*		Q86U17
	*Mus musculus*		Q8CIE0

For each sub-family of SERPINAs, are listed the species names, in case of multigenic families, the nomenclature of proteins and the accession numbers.

## Authors' contributions

PP and LB participated in the design of all experimental strategies employed throughout the research and performed the molecular genetic experiments. DD participated in the design of this study and carried out all bioinformatics and gene cluster organization analysis. AG carried out the phylogenetic analyses. XB participated in sequence analysis of *SERPINA3-7 *and *SERPINA3-8 *genes. SB and AO contributed greatly to the proteomic experiments. AM contributed to the finalization of the manuscript. All authors read and approved the final manuscript.
